# Exploring Protein-Peptide Binding Specificity through Computational Peptide Screening

**DOI:** 10.1371/journal.pcbi.1003277

**Published:** 2013-10-24

**Authors:** Arnab Bhattacherjee, Stefan Wallin

**Affiliations:** Department of Astronomy and Theoretical Physics, Computational Biology and Biological Physics group, Lund University, Lund, Sweden; University of California San Diego, United States of America

## Abstract

The binding of short disordered peptide stretches to globular protein domains is important for a wide range of cellular processes, including signal transduction, protein transport, and immune response. The often promiscuous nature of these interactions and the conformational flexibility of the peptide chain, sometimes even when bound, make the binding specificity of this type of protein interaction a challenge to understand. Here we develop and test a Monte Carlo-based procedure for calculating protein-peptide binding thermodynamics for many sequences in a single run. The method explores both peptide sequence and conformational space simultaneously by simulating a joint probability distribution which, in particular, makes searching through peptide sequence space computationally efficient. To test our method, we apply it to 3 different peptide-binding protein domains and test its ability to capture the experimentally determined specificity profiles. Insight into the molecular underpinnings of the observed specificities is obtained by analyzing the peptide conformational ensembles of a large number of binding-competent sequences. We also explore the possibility of using our method to discover new peptide-binding pockets on protein structures.

## Author Summary

The interactions between proteins play a crucial role for almost every undertaking of a cell. Many of these interactions are mediated by the binding of relatively short unstructured polypeptide segments, or peptides, in one protein to well-folded domains in other proteins. Such protein-peptide interactions have some interesting and special properties, e.g., promiscuity, which means many different peptide sequences are able to bind the same protein domain. Peptides also often exhibit structural flexibility even after binding a protein. These special properties make it desirable, but also challenging, to simulate protein-peptide binding in atomistic detail for many different peptide sequences. To this end, we have developed a computational algorithm that simultaneously explores the structure of protein-peptide complexes and the amino acid sequences of the peptide. In particular, our algorithm allows binding-competent peptide sequences to be generated in direct relation to their binding strengths. We also explored the possibility of using our method to locate new peptide-binding pockets on protein structures. Computational algorithms such as the one developed here may pave the way to reveal the full complexity of protein-protein interaction networks used in cells.

## Introduction

Protein-peptide interactions are involved in wide range of cellular processes and are more common than originally thought. Disordered peptide segments, often found within longer regions of disorder in proteins, typically undergo a binding-induced folding transition upon contact with a target molecule such that a specific structure is assumed [1]. It is not uncommon, however, that significant conformational diversity persists even after binding [2]–[4]. Disordered regions in proteins play pivotal roles in controlling cellular signaling networks [5], protein subcellular localization [6], [7], protein degradation [8], and post-translational modification [9], [10]. Remarkably, a recent estimate suggests that as much as around 40% of all links in protein interaction networks are due to binding of short peptide segments of around 3–10 amino acids in length to protein domains [11].

An apparently general property of protein-peptide interactions is their promiscuous nature, i.e., certain peptide positions contribute very little (or not at all) to the binding affinity, and thus can accommodate various amino acid types, while other positions require specific amino acid types for binding [12]–[14]. Indeed, many domain families recognize sets of peptide sequences conforming to particular amino acid patterns, or linear motifs. For example, SH3 domains bind sequences containing P-X-X-P where X is any amino acid and P is proline [12], and PDZ domains target short sequence patterns occurring at the extreme C-terminal end of proteins [15]. More than 100 such different linear motifs are known [16], however, many remain to be discovered [11]. Putative new linear motifs can be found by mining for overrepresented sequence patterns in evolutionarily related proteins [17] or in unrelated proteins sharing a common functional characteristic [18]–[20]. These methods are, however, limited by weak statistical signals, and cannot discover peptide segments involved in very few interactions or those not conforming to linear motifs. Subtle variations in specificity among domain members beyond a simple motif are crucial to their biological function [21], [22]. It is therefore of importance to understand the detailed molecular underpinnings of protein-peptide recognition. To this end, simulation methods at the atomic level have recently been employed, including different variants of docking [23]–[29], implicit- and explicit-water molecular dynamics [30]–[34], and Monte Carlo-based approaches [35]–[37].

**Figure 1:**
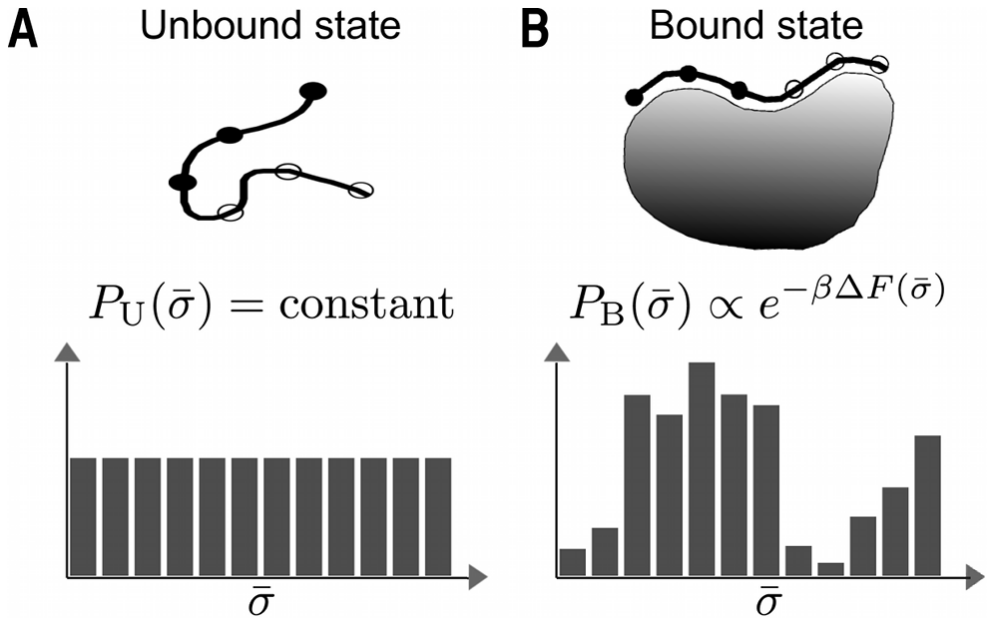
Schematic illustration of the computational peptide screening method. The method is based on simulating a joint probability distribution, 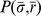, where 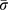 and 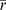 are amino acid sequence and chain conformation, respectively. Both conformational (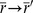) and “mutational” (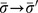) updates are performed as ordinary MC moves, subject to a Metropolis accept/reject question. Mutational updates are applied to a set of pre-defined variable amino acid positions on the peptide (open circles) while all other amino acids remain unchanged (filled circles). The procedure works in two steps. (A) In the first, iterative simulations of the unbound state (a free peptide) are performed creating a reference state where all 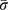
s occur with equal probability, i.e., the probability distribution 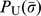 is flat. (B) In the second step, simulations of the protein-peptide bound state, B, are performed in which the distribution of 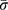 becomes skewed according to the Boltzmann weights 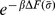, thereby favoring sequences with low binding free energies, 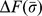. The probability distribution 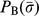 can be used to estimate relative 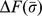-values among the different sequences 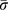 or give a representative view of the peptide-binding specificity of the protein.

Because of the promiscuous nature of protein-peptide interactions, determining peptide binding specificity profiles requires finding the binding free energy for a large number of different sequences. This can be computationally prohibitively expensive, especially since peptide chain entropy can contribute significantly to binding affinity [31]. In this work, we describe and test a theoretical framework for exploring, in an efficient and representative way, the combined sequence and conformational space of peptides interacting with a given peptide-binding pocket. In testing the method, we focus on 3 different PDZ domains with distinct peptide-binding specificity profiles. The method developed relies on the so-called multisequence Monte Carlo (MC) approach [38], [39] in which a joint probability distribution in conformation and sequence space is simulated. Updates in conformation and sequence are performed as ordinary MC moves and thereby put on an equal footing. In particular, this makes search through sequence space fast compared to calculating binding free energies for peptide sequences one after another. In our scheme, a representative sample of strongly binding peptide sequences can be obtained because the conditional probability distribution of sequences given bound peptide conformations becomes biased according binding free energy weights, as schematically illustrated in Figure 1. A major advantage of our method is that the underlying equilibrium conformational ensembles are readily available, which can provide insight into the interplay between specificity and the peptide conformational dynamics. We also explore the possibility of employing our method to the discovery of peptide-binding pockets, given only a protein structure as input.

## Methods

### All-atom computational model

All calculations in this work are performed using the model in Ref. [36]. It is an implicit-solvent model combining an all-atom representation of the protein chain with an effective energy function taking into account the major contributions of protein interactions, hydrogen bonding, electrostatic attraction, and the hydrophobic effect [40]. The model was developed and tested based on the folding of small peptides and proteins and thereafter adapted particularly for protein-peptide binding [35], [36]. The potential energy function can be decomposed into five terms,
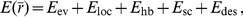
(1)representing excluded-volume interactions, local backbone interactions, hydrogen bonding, sidechain-sidechain interactions, and a backbone desolvation effect, respectively [36]. Because of the effective nature of the energy function, assigning a physical unit to the energy 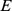 is not straightforward. We therefore use dimensionless units to express 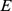 and 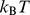, where 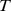 is the temperature. All bond lengths and angles are kept fixed at values derived from a statistical analysis of protein structures in the Protein Data Bank [41]. In addition, some torsional angles, such as the peptide bond angle 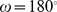, are kept fixed at “ideal” values. Therefore, the degrees of freedom of the model are a set of torsional angles and overall chain orientations. More precisely, a conformation of one or more protein chains, 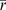, is determined by the backbone torsional angles, 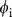, 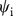, a set of sidechain torsional angles, 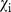, for each amino acid i, and the overall rotational and translational orientation of each chain.

### Protein-peptide binding specificity

Consider the interaction between a protein structure and a 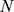-amino acid peptide with sequence 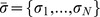 where 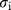 is the amino acid type of position i, and denote the chain conformation of the protein and peptide by 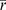. In principle, a complete description of the peptide-binding specificity of the protein means finding the binding free energy 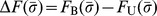, where 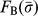 and 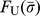 are the free energies of the bound (B) and unbound (U) states, respectively, for all possible 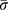. This definition of binding free energy requires classifying conformations 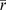 as either B or U which can be done using some geometric criterion, such as the closeness of the peptide backbone to the peptide-binding pocket.

Binding free energy calculations are computationally intensive because they in principle require a full exploration of 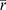-space. For instance, the binding free energy of particular peptide at temperature 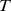 can be calculated using
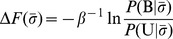
(2)where 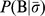 and 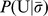 are the probabilities of populating B and U, respectively, for a given peptide sequence 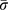 at temperature 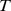, 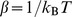, and 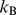 is Boltzmann's constant. Therefore, determining 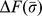 for many 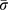 in a sequential manner is time consuming. As an alternative, we develop here a method that in a single run generates sequences from the probability distribution
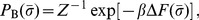
(3)where 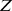 is a normalization constant. Hence, rather than searching for a single optimally binding peptide, our method aims to “screen” for peptide sequences with low 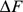 in a controlled manner thereby providing a representative picture of the peptide-binding specificity.

### Multisequence Monte Carlo method for protein-peptide binding

The approach in this work for generating sequences according to the distribution in Equation 3 is based on the multisequence Monte Carlo method [38], [39], meaning it relies on simulations of the joint probability distribution
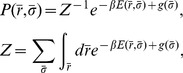
(4)where 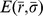 is the potential energy of a conformation 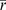 with sequence 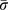, and the sum and integral are taken over all 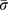 and 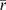, respectively. Practically, this means designating a set of amino acid positions on the peptide as *variable*, for which the amino acid type is allowed to change dynamically through MC updates (see below for details). The parameters 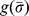 are important as they control the marginal distribution
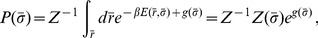
(5)where 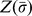 is the canonical partition function for sequence 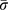 at temperature 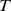. We now make use of the division of 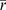-space into B and U states, such that 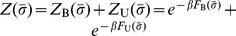. This allows us to construct the probability distribution
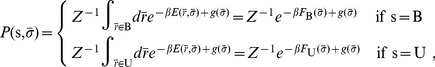
(6)which can be used to construct a ratio,
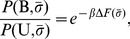
(7)determined by 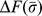 and hence independent of the parameters 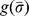. Equation 7 shows that it is in principle possible to replace the sequential calculation of binding free energies 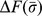 for many 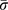, by a *single* multisequence simulation of the distribution in Equation 4 and measuring the probabilities 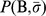 and 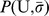. Such an approach is possible but it also has practical limitations. The number of 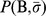 and 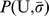 quantities to be estimated grows exponentially (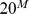) with the number of variable positions, 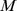, meaning the approach is limited to very small 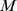. The approach does in principle not depend on the parameters 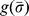 but in practice they would need to be carefully chosen to achieve sufficient sampling of sequence space, even for small 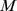.

### Computational peptide screening method

We do not pursue free energy calculations based directly on Equation 7 in this work. However, we take it as a starting point for developing our peptide screening method. First, we restate the probabilities in Equation 7 using Bayes' theorem,
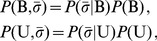
(8)where 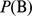 and 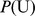 are the total probabilities of occupying B and U (regardless of 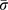), respectively, and 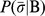 and 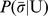 are conditional probabilities. Second, we make the choice 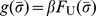. This means that the distribution 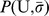, and hence 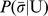, becomes flat (cf. Equations 6 and 8). We then obtain
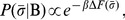
(9)and we can make the identification 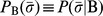 (see Equation 3). To simplify our notation, we also put 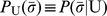.

It is important to note that the conditional probabilities 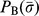 and 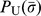 are computationally convenient quantities because they do not depend on states 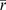 outside B and U, respectively. They can be obtained from separate multisequence simulations where 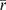 is restricted to B and U. We can now summarize our peptide screening method as a two-step strategy, illustrated in Figure 1:

1. *Unbound state simulation*. Determine 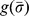 parameter values such that all sequences 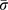 occur with equal probability in a multisequence simulation of U, i.e., such that 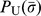 becomes flat.
2. *Bound state simulation*. Using the obtained 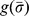, perform a multisequence simulation of B. The generated sequences 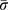 will become distributed according to the Boltzmann weights 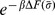.

### Unbound state approximation

To further simplify our implementation of the above strategy we make the approximation that U consists of a free protein and a free peptide, without any interaction. There are then two contributions to the unbound state free energy 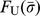, a 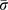-independent contribution from the protein, 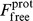, and a 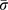-dependent contribution from the free peptide, 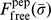. We can ignore the quantity 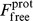, putting 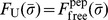, because 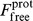 does not impact the distribution
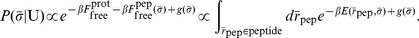
(10)Hence, in calculations of the unbound state, we can rely on multisequence simulations of a free peptide chain ignoring the protein.

### Linear model of the unbound state

A remaining question in implementing the strategy outlined above is how to determine the parameters 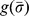, such that they approximate well 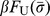. We find that a simple linear form,
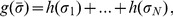
(11)where 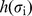 depends on amino acid type, is sufficient to achieve a good approximation. The 20 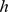-parameters can be interpreted as the contributions made to 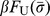 by the various amino acid types. This can be seen by considering the case in which the 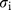
s are independent variables. In such a case, the unbound state free energy can be decomposed into position-independent contributions 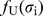, i.e., 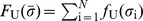, and the conditional probability distribution of 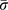 given the unbound state can be written 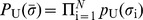, where
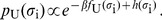
(12)Hence, the choice 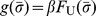 amounts to setting 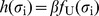. A good set of 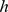-parameters can be found by iterative multisequence simulations of U (a free peptide) in which a flat distribution in sequence space, 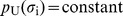, is eventually obtained. As seen from Equation 12, by measuring the probabilities 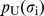 in simulations with an initial 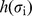 set, an improved set of values can be obtained by setting 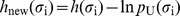.

Using this procedure, we have determined 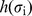 parameters for 3 short peptides which provides approximately flat 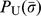 distributions (see Figure S2 in Supporting Information). In particular, this shows that, despite the simplification, a linear approximation is sufficient to achieve 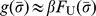 to a reasonably good approximation. This is important for the peptide screening method because it underlies the accuracy of Equation 9 which assumes 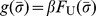. Errors in the approximation to the unbound state free energy will directly affect the conditional distribution, 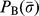. More precisely, if 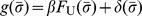, then
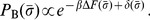
(13)The approximation errors 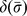 are generally not possible to determine individually due to the size of the sequence space. An indication of the size of the errors can, however, be obtained from Figure S2. It shows that for the probability distributions of different amino acid types taken over all variable positions, the deviations are at most around 10%. Similar deviations from the desired Boltzmann distribution (see Figure 1B) for 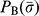 should be expected. We also note that more elaborate approximations to 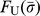 could easily be implemented, e.g., a position-dependent linear approximation with 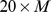 free parameters rather than the 20 parameters in Equation 11.

Errors introduced by the linear approximation on U would not impact free energy calculations performed using Equation 7, because this ratio is independent of the choice of 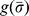. Choosing 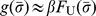 would nonetheless be a suitable choice for this method too, as a way to achieve good sequence space sampling.

### Monte Carlo updates

The distribution in Equation 4 is realized through multisequence MC simulations. In these simulations, two different types of MC updates are included. Updates of the first type are conventional conformational updates (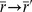) and include pivot moves, 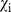-angle rotamer turns, and rigid body rotation and translations, as described in previous work [35], [36]. The second type of updates produces changes to the amino acid sequence of the peptide (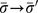). These “mutational moves” are subject to an ordinary Metropolis accept/reject question, i.e., the new sequence is accepted with probability 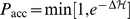, where 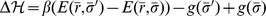. Proposed sequences 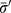 are obtained by randomly picking a variable peptide position i and a new amino acid type, 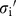. Thereafter, the peptide chain is re-built using the current 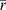, i.e., the set of 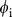-, 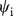-, and 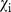-angles, and the new energy 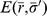 calculated. A complication is that the number of actual degrees of freedom for different amino acid types differ. This can be handled by formally including two backbone angles, 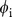 and 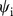, and 5 side-chain angles, 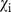, as degrees of freedom for every variable amino acid position (7 is the maximum number of internal degrees of freedom for a residue in our model, occurring for lysine). For example, the geometry of an alanine residue is determined by two 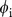, 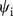 angles and a 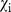 angle. This means that the potential energy 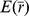 is independent of the remaining 4 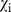 angles, which will therefore quickly tend towards a uniform distribution. In a proposed mutation to an amino acid with additional (actual) degrees of freedom at position i, such as serine, the new amino acid will inherit the two 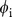, 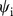 angles and all 5 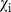 angles, which will determine its geometry. That detailed balance is indeed maintained by this scheme can be explicitly seen by comparing multisequence and a set of separate ordinary simulations of short peptides (see Figure S3 in Supporting Information).

### Protein domains

The 3 peptide-binding proteins considered in this work are the 3rd PDZ domain of PSD-95, the 6th PDZ domain of GRIP1, and the PDZ domain of PICK1, which we refer to throughout the text as PSD95, GRIP1, and PICK1, respectively. Structures of peptide-bound complexes have been determined with X-ray crystallography for PSD95 (PDB id 1BE9) [42] and GRIP1 (1N7F) [43], and with NMR for PICK1 (2PKU) [44], [45] (see Figure S1 in Supporting Information), with peptides sequences KQTSV, ATVRTYSC, and ESVKI, respectively.

### Monte Carlo simulations

In order to test our peptide screening procedure (Figure 1), we perform also “fixed-sequence” simulations for comparison, following our earlier protocol [35], [36]. This procedure explores the interaction between a given protein structure and a given peptide sequence in a straightforward way. The protein is kept close to an experimentally determined native structure through constraints on the 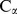-atoms, leaving some backbone flexibility and complete sidechain flexibility. The peptide chain, by contrast, is left without constraints such that it can explore the entire protein surface. The protein and peptide chains are contained within a cubic box (side *L* = 50 Å) with periodic boundary conditions, corresponding to an effective concentration of 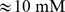. To achieve an equilibrium picture of the interaction, the (dimensionless) simulation temperature is set such that both binding and unbinding events occur. In the present study, 10 independent fixed-sequence simulations of at least 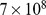 MC steps were performed at 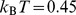 for each of the 9 PSD95-peptide pairs taken from Ref. [46].

Our peptide screening simulations (Figure 1B) differ from these fixed-sequence simulations in two ways. First, the peptide chain is restricted to the peptide-binding pocket of the protein using a constraint on the 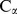-atom of the peptide C-terminal residue. This constraint is loose enough to still allow binding and “unbinding” of the peptide such that conformations in the bound state can be fully explored (for details, see section Peptide-binding pocket constraint). Second, in addition to the conformational MC updates for the protein and peptide chains, mutational updates are applied to the variable positions of the peptide (see above).

For PSD95, peptide screening simulations were performed with the peptides KKETE-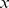 and KKE-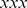, where 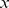 indicates a variable amino acid position (derived from the sequence KKETEV which has been identified as a high affinity binder for PSD95 [46]). For GRIP1 and PICK1, simulations were performed for ATVRT-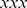 and ES-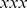, respectively. For each system with 3 variable positions we performed 20 independent runs, and for KKETE-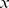 3 independent runs were performed. All trajectories were at least 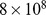 MC steps in length. The simulations were performed at 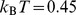, 0.55, and 0.51, for PSD95, GRIP1, and PICK1, respectively. These values were determined previously as midpoint temperatures for the different PDZ domains with their respective peptide ligands [36]. Simulations of the unbound state (step 1, Figure 1A) were performed for free peptide chains at the same respective temperatures. All multisequence simulations were initiated with 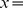
alanine at the variable positions.

### Peptide bound state

To monitor binding of the peptides in our simulations, we use a root-mean-square distance between the native and model peptide coordinates, 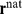 and 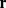, i.e.,
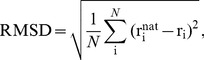
(14)where the sum goes over the 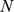 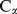-atoms of the peptide. The native peptide coordinates are taken from an experimentally determined structure (see Protein domains above). The peptide bound state (B) is defined as RMSD<6 Å, following Refs. [35], [36].

### Peptide-binding pocket constraint

To spatially constrain the peptide chain close to the peptide-binding pocket we use a simple constraint energy term, 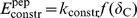, where 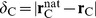 is the deviation of the peptide C-terminal 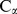-atom position, 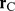, from its position in the experimentally determined structure, 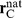. The function 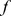 is piecewise linear such that 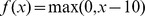. This means that if the peptide C-terminal end moves more than 10 Å from its position in the native structure, there will be an energetic penalty. The constraint term was chosen in order to enhance sampling of the peptide bound state, without forbidding important bound state structures. The strength of the term is set to 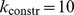.

## Results/Discussion

In order to realize the computational peptide screening method imagined in Figure 1, a prerequisite is that relative binding free energies for different peptide sequences can be reasonably well estimated. We therefore start by testing our all-atom computational model for protein-peptide binding for predicting binding free energies on one of our 3 test domains. Second, we test the soundness of the developed screening method by comparing with the same binding free energy data. Third, we test the ability of the method to reproduce more generally the binding specificity profiles of the 3 domains and link them to conformational preferences of the peptide chain in the bound state. Lastly, we attempt “unrestricted” peptide screening in which the peptide is allowed to search freely the protein surface. Such an approach could potentially be used to locate new peptide-binding sites on protein structures.

### All-atom computational model for protein-peptide binding

Previously, we have developed a MC-based approach for protein-peptide binding [35], [36]. In this approach, the peptide is left free to explore the protein surface and relatively long simulations are performed such that a representative conformational ensemble can be obtained, including both bound and unbound states. The underlying all-atom model is taken from folding studies of proteins and was tested on a larger set of PDZ domains and peptides, particularly by comparing minimum-energy conformations with experimental structures of the protein-peptide complexes. In 8 out of 11 cases, the minimum-energy structures were within a root-mean-square distance RMSD (see Methods) of 6 Å from the experimental structures [35]. The method has also been used to study details of the peptide binding process by exploring the binding free energy landscapes for PDZ domains of different specificity classes [36].

We turn now to the ability of our model to quantitatively reproduce experimental binding affinity data. To this end, we use a study by Spaller *et al.* [46] in which isothermal titration calorimetry was used to determine binding affinities for the domain PSD95 and a number of peptide sequences under identical conditions. Of the peptides in Ref. [46], we focus on the 6-amino acid peptide KKETEV, a known high-affinity binder for PSD95, and 8 variants with modifications in either 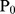 or 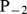; for PDZ peptide ligands, the C terminal position is denoted 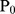 and the positions immediately upstream are 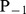, 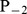, 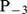, etc. Using our procedure [35], [36], (see also Methods) we performed simulations of the interaction of each of these 9 peptides and PSD95. Binding free energies can be calculated in a straightforward way using Equation 2. To this end, we define the bound state, B, as peptide conformations with RMSD<6 Å, again following Refs. [35], [36]. The experimentally measured dissociation constants, 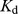, differ by approximately two orders of magnitude for the 9 peptides sequences, from 1.9 to 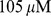 [46].

**Figure 2:**
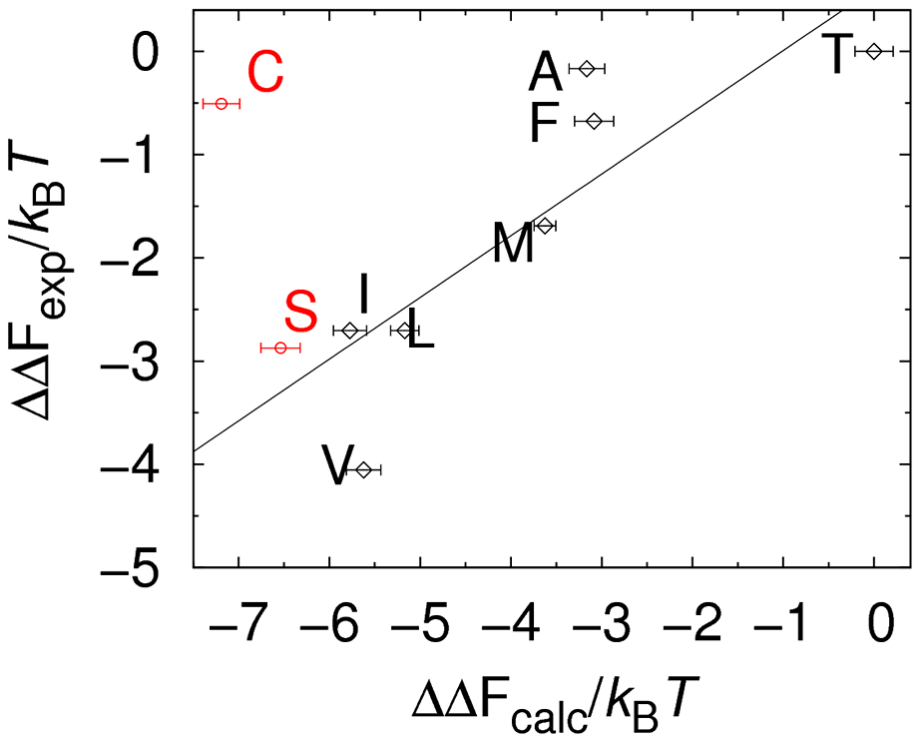
Comparing experimental and calculated relative binding free energies. As a quantitative test of the all-atom model [36] used in this work, we calculate binding free energies, 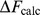, for the protein PSD95 and 9 different peptide sequences. The 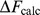 values were obtained from protein-peptide binding simulations performed separately for each PSD95-peptide pair according to our previous protocol [35], [36] (see also Methods) and using Equation 2. The peptide sequences considered are derived from KKETEV (black diamond) and are either 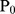 -variants, KKETE-[I/L/M/F/A/T], (black diamonds) or 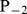 -variants, KKE[S/C]EV (red circles). All simulations were performed at 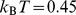 and standard errors were estimated from 10 independent runs. Experimental binding free energies, 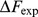, are taken from Ref. [46]. Both 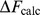 and 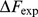 values are shown relative to the weakest binding peptide. The solid line represents the best linear fit, exluding KKECEV, and the correlation coefficient is 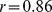.

In Figure 2, experimental and calculated binding free energies are compared. There is a reasonably good agreement between the two sets of data, with the exception of one of the 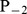 -variants which is predicted to bind too strongly. Excluding this outlier, the correlation is 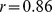. It should be noted that the comparison in Figure 2 involves relative binding free energies. Absolute binding free energies obtained from Equation 2 are generally different from those measured by Spaller *et al.* [46]. The reason is that our simulations are performed at a computationally convenient temperature where equilibrium can be reached, i.e., where both binding and unbinding are observed multiple times in a trajectory. The binding affinities obtained are relatively low (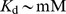) meaning that the simulation conditions used, 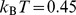, correspond to a higher temperature than the 298 K used in the experiments [46]. For the same reason, although the correlation between experimental and calculated binding free energies is good, the ranges observed are slightly different (approximately 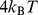 and 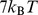, respectively). The disagreement for the outlier sequence KKECEV, however, cannot be explained by temperature differences but rather indicates a limitation of our model in capturing all relevant energetics of the binding process. For class I PDZ domains, which includes PSD95, interactions at 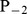 involve rather subtle intermolecular sidechain-sidechain hydrogen bonding (between the serine or threonine at 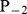 and a histidine on the 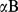-helix of the PDZ domain [15]) that might not be captured entirely by our model. Overall, the result indicate that our model captures well variations in binding free energies among 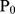 -variants while there are some limitations for 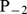 -variants.

### Testing the computational peptide screening method

We now turn to our computational peptide screening method. As illustrated by Figure 1, the method works by performing multisequence MC simulations in two steps. In these simulations, interlaced updates in conformational space and peptide sequence space (for variable peptide positions) are performed as ordinary MC updates. The first step involves iterative simulations of the unbound state (a free peptide chain) such that a reference state is created where all peptide sequences occur with roughly equal probabilities. In the second step, simulations of the peptide-bound state are performed where, by contrast, peptide sequences 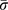 will be generated in a biased way according to the weights 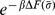. The theoretical background is described in detail in Methods.

**Figure 3:**
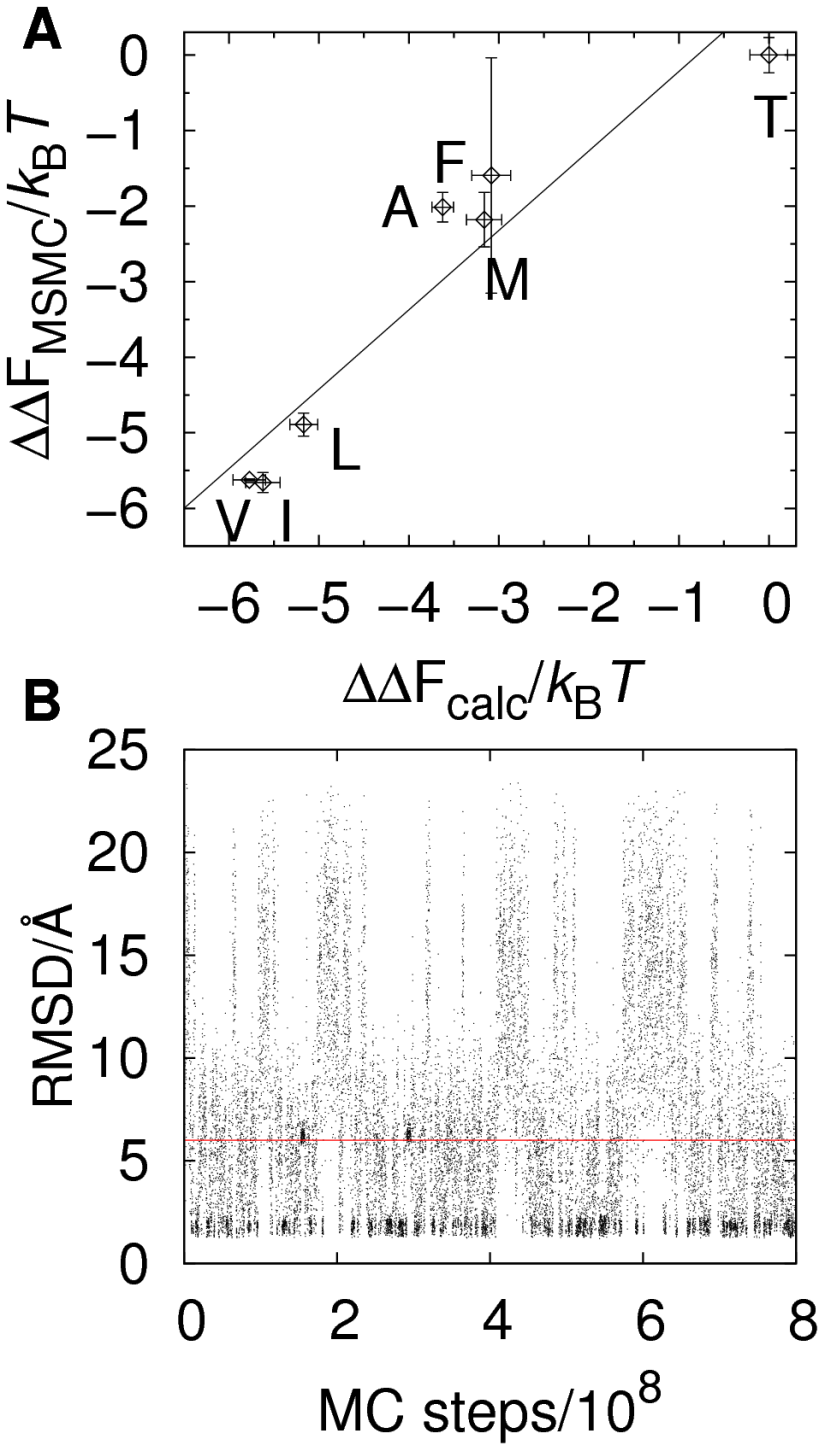
Relative binding free energies from computational peptide screening. We applied the peptide screening method to the protein PSD95 with the peptide KKETE-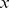, where 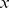 denotes a variable amino acid position. Relative binding free energies, 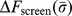, for the different possible peptide sequences 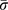, were estimated from the distribution 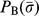 obtained in the second step of the screening procedure (see Figure 1) and using the relation 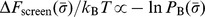. (A) Comparison between 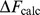 and 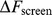 values for the 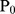-variants in Figure 2. The correlation coefficient is 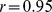. Standard errors for 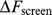 are estimated from 3 independent screening runs. As in Figure 2, the binding free energies are shown relative to the weakest binding peptide. (B) Example of a peptide screening run of PSD95 showing the evolution of RMSD, which measures the structural similarity of the peptide chain to the experimental structure, as a function of the number of elementary MC steps. The definition of bound state, RMSD<6 Å, is indicated by a horizontal line.

As an initial test, we consider again PSD95 and screen the PDZ peptide-binding pocket using the peptide sequence KKETE-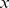, where 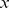 indicates a variable amino acid position. An example of a trajectory of the bound state B is shown in Figure 3B. Because the distribution of generated sequences is known (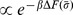), the frequency of occurrence of different amino acid types at position 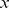 can be used to estimate the relative binding free energies for the 20 different sequences (i.e., KKETEG, KKETEA, KKETEV, etc.). These screening results can be directly compared with our results above, obtained for full protein-peptide simulations performed separately for different sequences. Our screening-derived binding free energies, 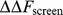, correlates well (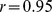) with our previously obtained 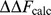 values, as shown in Figure 3A. There are two approximations inherent to the peptide screening method. First, it is assumed that the unbound state consists of a free protein and a free peptide, without any interaction. Second, a linear approximation of the unbound state free energy is applied (see Methods for details). The agreement between the two different sets of results for PSD95 shown in Figure 3A means, in particular, that the approximations underlying the screening method do not strongly impact the results.

### Exploring peptide binding specificities

We now employ our screening method with the aim of more generally characterizing the peptide-binding specificity of a given protein domain. To this end, we apply the screening approach as in the previous section but now allow additional variable amino acid positions such that the main specificity-determining region of the peptide is covered. We focus on three different domains, PSD95, GRIP1, and PICK1, each having a different specificity profile. PSD95 and GRIP1 are representative members of class I and II PDZ domains binding peptides with the sequence pattern 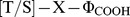 and 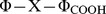, respectively, where X is any residue, 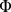 is a hydrophobic residue, and COOH represents the peptide C terminus [15], [47]. PICK1 is a domain with dual specificity, meaning that it binds peptides exhibiting either class I or II sequence patterns. We perform simulations in which 3 peptide positions are treated as variable, 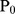, 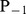, and 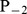.

**Figure 4:**
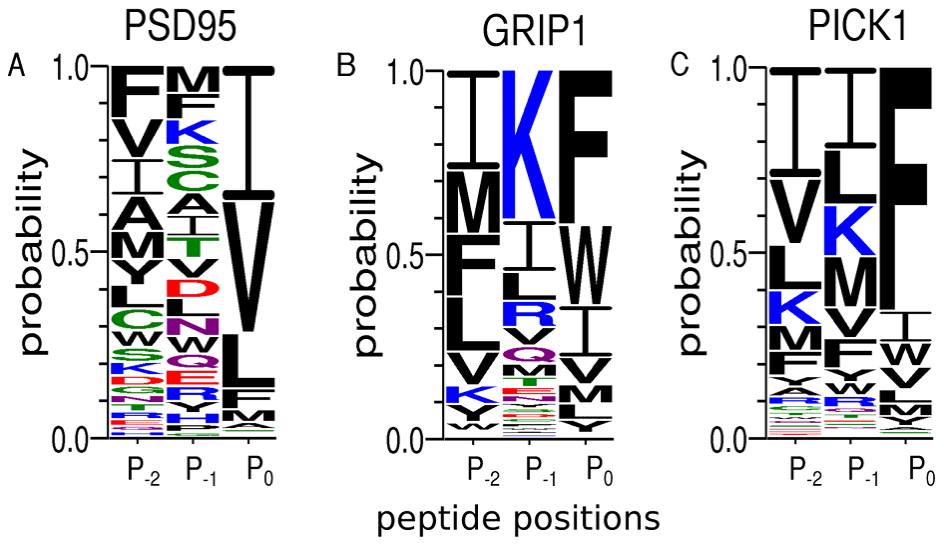
Peptide-binding specificity profiles from computational peptide screening. We applied our peptide screening method to the proteins (A) PSD95, (B) GRIP1, and (C) PICK1 with the peptides KKE-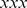, ATVRT-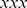 and ES-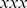, respectively, where 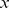 indicates a variable amino acid position. As illustrated in Figure 1, the obtained probability distributions 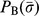 represent the peptide-binding specificities of the proteins. Shown are “one-dimensional” specificity profiles for the 3 variable peptide positions, illustrated as weblogos [48]. At a given peptide position i, the letter height is determined by 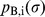, i.e., the probability of observing the amino acid type 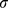 at position i. The color scheme is as follows: hydrophobic (A, V, L, I, P, W, F, M, Y) black, polar (G, S, T, C) green, neutral (Q, N) purple, basic (K, R, H) blue, and acidic (D, E) red.

To identify possible binding motifs for a particular domain, we use weblogos [48] to represent the obtained conditional distribution of sequences, 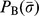. It is clear from Figure 4 that our result for GRIP1 is consistent with a class II domain, as expected from experimental data. The situations is less straightforward for PSD95. The position 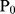 is occupied mainly by hydrophobic residues, particularly I, V, and L, and 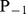 samples amino acid types almost uniformly. This in line with experimental results which have identified 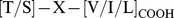 as the linear motif for PSD95 [46]. For 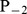, our simulations give only a weak signal for T/S which is likely due to the limitation of our model in capturing fully the binding energetics for this position for class I domains, as discussed above. It is interesting, however, that T7 phage display experiments [49] produced instances of hydrophobic residues at 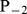, particularly I and M, giving some support to our result in Figure 4A. This is also in line with the notion that the classification of PDZ domains is not strict and cross-interactions with other ligands are possible [22]. For PICK1, we find a specificity profile more closely related to class II rather than class I (see Figure 4C). This unexpected result suggests that PICK1 might be class II dominant despite its dual specificity nature. Further experiments will be needed to explore this possibility. It is at least partially supported by the study of Madsen *et al.* [50] where, using an assay based on fluorescence polarization, it was found that PICK1 showed a higher affinity for a class II than class I peptide. We also note that our screening method predominantly produce F residues at 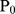 which is contrary to Ref. [50], [51] where a preference for smaller hydrophobic residues was seen.

**Figure 5:**
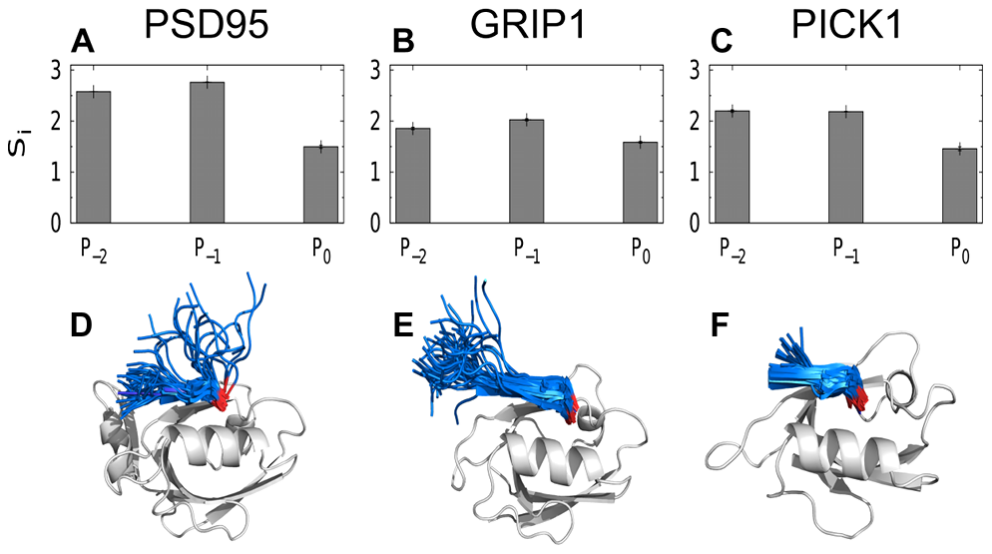
Interplay between peptide binding specificity and structural heterogeneity. A useful feature of the peptide screening method is that the underlying joint probability distribution 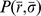 (see Figure 1) can provide further insight into the structural underpinnings of specificity. Shown is a simple analysis of the specificity profiles of PSD95, GRIP1, and PICK1 in Figure 4. (A–C) The degree of sequence randomness at different peptide positions i, as measured by the sequence entropy 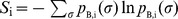, where 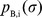 is defined as in Figure 4 and the sum goes over all 20 different amino acid types 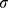. For reference, we note that for a position in which all amino acid types occur with equal probability, 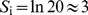. (D–F) Superposition of a random sample of bound state conformations 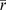 with various peptide sequences 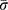, in ribbon representation (peptides shown in blue and PDZ domains in grey). For clarity, only single structures of the PDZ domains are shown. We find that the relatively larger structural heterogeneity at 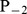 for peptides bound to PSD95 is connected to a higher 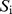. The C-terminal amino acid, 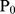, (red) is tightly bound to the peptide-binding pocket in all 3 cases and this feature is conserved across different binding-competent sequences.

The profiles in Figure 4 indicate that overall sequence variations are tolerated to different extents in the 3 different peptide positions. This is quantified in Figure 5, showing the sequence entropy, 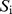, for the different amino acid positions. What is the reason for these differences? We note that 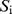, which measures the degree of sequence randomness, is smallest for 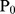 and largest for 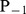 for all 3 domains. This is consistent with the general property of PDZ domains allowing only hydrophobic amino acids at 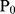 while accommodating (mostly) any amino acid type at 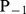. Figure 5A shows that PSD95 exhibits a relatively high 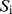 also for 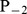. This is likely due to a limitation of our model to fully capture the T/S preference at this position, as discussed above. The slight preference for hydrophobic amino acids at 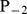 is nonetheless interesting given the experimental support [49] for this observation. How can such atypical hydrophobic amino acids at 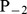, in some cases, be accommodated by a class I domain for which the recognized linear motif typically follows 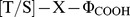
? In Figure 5D–F, we illustrate representative ensembles of bound peptide conformations for all three domains (regardless of peptide sequence, 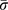) obtained from our screening simulations. PSD95 differ from the other two domains in that it displays a greater structural diversity of the peptide ligand. Such relatively major conformational flexibility is not uncommon for small ligands in complexes [52] and has been observed by our group previously for this PDZ domain [36]. In a somewhat simplified picture of PDZ-peptide binding, the bound state can be seen as a combination of two binding modes, where the peptide binds the domain either in a tight way, involving both 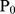 and 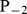, or in a looser way, involving only 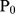 (see Figure 5 and Figure S4 in the Supporting Information). This observation is in line with recently determined X-ray structures of class I PDZ domain-peptide complexes in which the peptides bind their respective domains mainly through 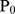 and directed roughly perpendicular to the domain surface [53]. It is possible therefore that hydrophobic amino acids might be allowed at 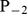, in particular cases where the bound state include such “perpendicular” peptide conformations. Nonspecific hydrophobic contacts between 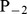 sidechains and the domain surface might contribute to the stability of the complex. Such a picture would indeed explain the occurrence of some hydrophobic amino acids at 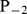 both in our results and in phage-display experiments performed on PSD95 [49].

### Discovery of peptide binding sites?

We have shown above that our peptide screening method can describe the gross features of the peptide binding specificities for a set of protein domains. The second step in our strategy (Figure 1B) involves multisequence simulations in which the peptide chain is artificially kept close to the peptide-binding pocket using a spatial constraint, in order to enhance the sampling of the bound state, B. Can the spatial constraint on the peptide be relaxed? The question is of interest because, if it turns out to be feasible, it opens up for using our screening method as a way to discover peptide-binding pockets on proteins, based on a 3-dimensional structure alone. To investigate the possibility for using our screening method for such structure-based binding-site discovery, we perform simulations of PSD95 following essentially the strategy in Figure 1, but with the difference that the peptide is left entirely unrestricted in the second step, i.e., it is free to diffuse in the simulation box and thus allowed to bind anywhere on the protein surface. Moreover, we make the 5 most C-terminal positions, 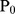 to 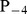, variable. This approach is therefore truly unbiased in the sense that no prior knowledge is built in of either (1) the peptide-binding pocket on the protein or (2) which peptide sequences are binding competent.

**Figure 6:**
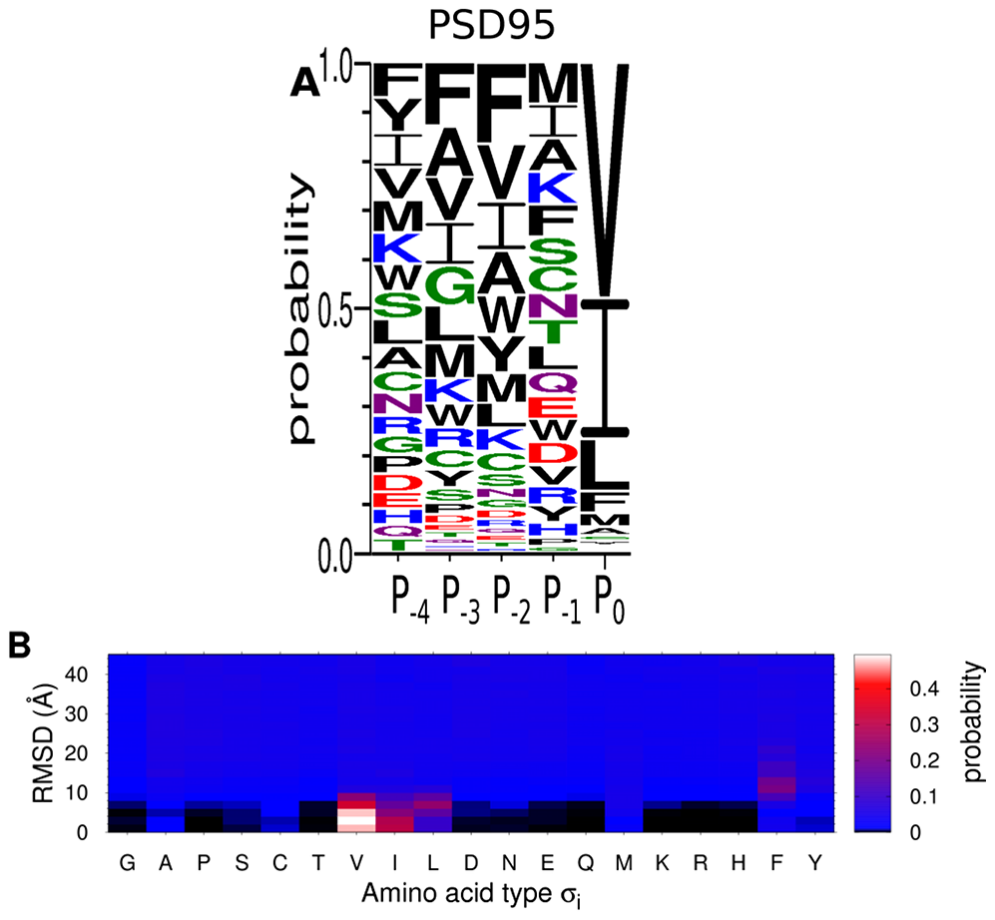
Using computational peptide screening for peptide-binding site discovery. We tested a modified version of the screening procedure in which the peptide is free to search the entire protein surface. This approach was applied to the protein PSD95 with the peptide G-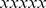 (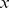 indicates a variable position), at 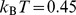. (A) Peptide-binding specificity profile determined as previously for the 5 variable positions, i.e., using the conditional probability distribution of 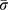 given the bound state, 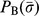. The letter color scheme is same as in Figure 4. (B) Probability distributions of 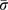 given different values of RMSD, 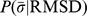, for the C-terminal peptide position, 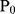. Far from the binding pocket (high RMSD), all amino acid types are visited roughly uniformly whereas close to the binding pocket (low RMSD) the distribution becomes skewed towards strongly binding sequences.

Figure 6 shows the results of these unbiased peptide screening simulations. In Figure 6B, we show a probability distribution in terms of RMSD and amino acid type, 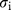, for 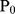. The distribution should be interpreted such that, for a given RMSD, it gives the probability for the occurrence of various amino acid types at 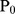. For example, at high RMSD values (>10 Å), the probability is roughly uniform (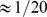), indicating that the peptide behaves as if in the unbound state. In particular, this means that peptide does not attach to surface regions other than the PDZ peptide-binding pocket despite the search through peptide sequence space. The picture changes drastically when the peptide is close to the peptide-binding pocket (low RMSD), in which the sequence distribution becomes skewed, particularly towards V, I, and L. In fact, the binding specificity profile for 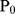, 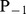, and 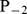, constructed using the same bound state definition as before (RMSD<6 Å), is highly similar to the one obtained previously using the “restricted” screening simulations (cf. Figure 4A and Figure 6A). The consistency of these results suggests that our peptide screening method might be able to function as a tool to identify peptide binding sites on protein structures and at the same time provide a rough estimate of the their peptide-binding properties.

### Summary and conclusions

We have developed an equilibrium MC-based method for characterizing protein-peptide interactions. The method samples jointly the peptide sequence and conformational spaces in a single run. In particular, this strategy makes search through sequence space computationally efficient and allows relative free energies to be estimated for a large number of peptides. In this work, we explored possible applications and used 3 different PDZ domains, with different peptide-binding specificities, as a test case. The peptide screening method relies on two approximations on the unbound state which are found not to impact the results significantly. Rather than measuring relative populations of bound and unbound states for many different peptide sequences, the method relies on measuring a conditional probability distribution of the sequences in a single run. Using this aspect of the method, we found good agreements with both full-scale protein-peptide binding simulations performed separately for each sequence as well as with experimental results. We also obtained specificity profiles for each of the 3 domains and compared with the experimentally known profiles, with a good overall agreement. An advantage of the method is that conformational ensembles are readily available for analysis, for visited sequences, which can reveal the interplay between binding specificity and conformational flexibility of the peptide chain. Finally, we explored the possibility of using the screening procedure for discovering new peptide-binding pockets on protein structures, with encouraging results.

## Supporting Information

Figure S1 **Experimental structures of the PSD95, GRIP1, and PICK1 domains in complex with peptide ligands.** Visualization of the X-ray structures of (A) PSD95 [42] and (B) GRIP1 [43], and the NMR structure of (C) PICK1 [44]. The peptide ligands have the sequences KQTSV, ATVRTYSC, and ESVKI, respectively, and are shown in stick representation (deep blue, except the C-terminal amino acids shown in red). The PDZ domains are shown in ribbon (light blue). The image was created using the PyMol molecular visualization program.
(TIFF)

Figure S2
**Multisequence Monte Carlo simulations of free peptide chains.** The first step of the peptide screening strategy (see Figure 1A) requires obtaining a uniform distribution of sequences, i.e., 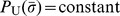. Multisequence simulations were performed of the isolated peptides KKE-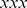 (PSD95), ATVRT-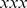 (GRIP1), and ES-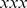 (PICK1), where 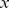 represents a variable amino acid position, at 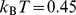, 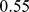, and 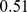, respectively. The figure shows probability distributions 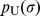 in amino acid type 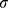 taken over all 3 variable positions. To achieve roughly flat distributions, i.e., 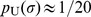, sets of 20 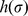 parameters were determined separately for each peptide by an iterative procedure, as explained in the text.(TIFF)

Figure S3
**Detailed balance in all-atom multisequence Monte Carlo simulations.** To test the soundness of the proposed method, we performed multisequence simulations of the peptide A-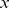-A, where 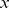 is a variable amino acid position, at 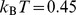. These simulations amounts to calculating the thermodynamic behavior for the 20 different variants 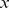 in a single run. For comparison, therefore, we perform ordinary MC simulations separately for each of the 20 tripeptides AGA, AAA, AVA, etc., at the same temperature. We test the consistency of the two set of results by comparing the probability distributions 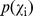 for different sidechain rotamer angles, 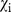. The figure shows 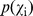 for (A) 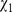, (B) 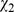, and (C) 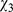 for valine, obtained from the “fixed-sequence” simulation (FSMC) of the tripeptide AVA and the multisequence MC simulation (MSMC) of A-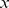-A, with 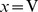. The consistency of the results confirms that the multisequence simulation samples the correct thermodynamic distribution.(TIFF)

Figure S4
**Conformational diversity in the PSD95 peptide-bound state.** Superposition of bound conformations from peptide screening simulations of PSD95, sub-grouped into peptides conformations with (A) 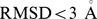 and (B) 3 Å<RMSD<6 Å, respectively.(TIFF)
