# Supplementary figures and images for: Exploring Protein-Peptide Binding Specificity through Computational Peptide Screening
Source: PLoS Comput Biol. 2013 Oct 24;9(10):e1003277. doi: 10.1371/journal.pcbi.1003277 (PMC3812049; doi:10.1371/journal.pcbi.1003277)

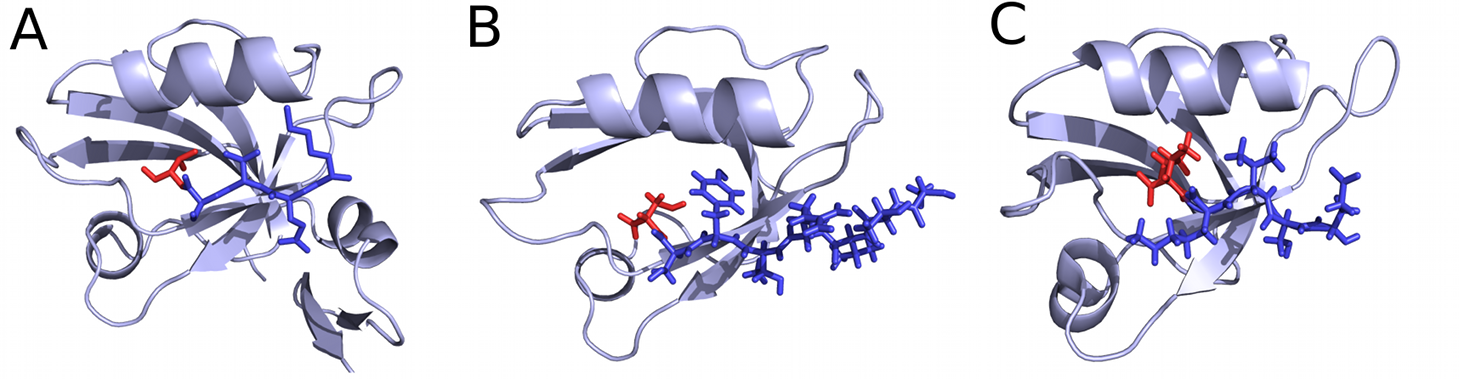

Supplement: Figure S1 — Experimental structures of the PSD95, GRIP1, and PICK1 domains in complex with peptide ligands. Visualization of the X-ray structures of (A) PSD95 [42] and (B) GRIP1 [43], and the NMR structure of (C) PICK1 [44]. The peptide ligands have the sequences KQTSV, ATVRTYSC, and ESVKI, respectively, and are shown in stick representation (deep blue, except the C-terminal amino acids shown in red). The PDZ domains are shown in ribbon (light blue). The image was created using the PyMol molecular visualization program. (TIFF) [file pcbi.1003277.s001.tiff]

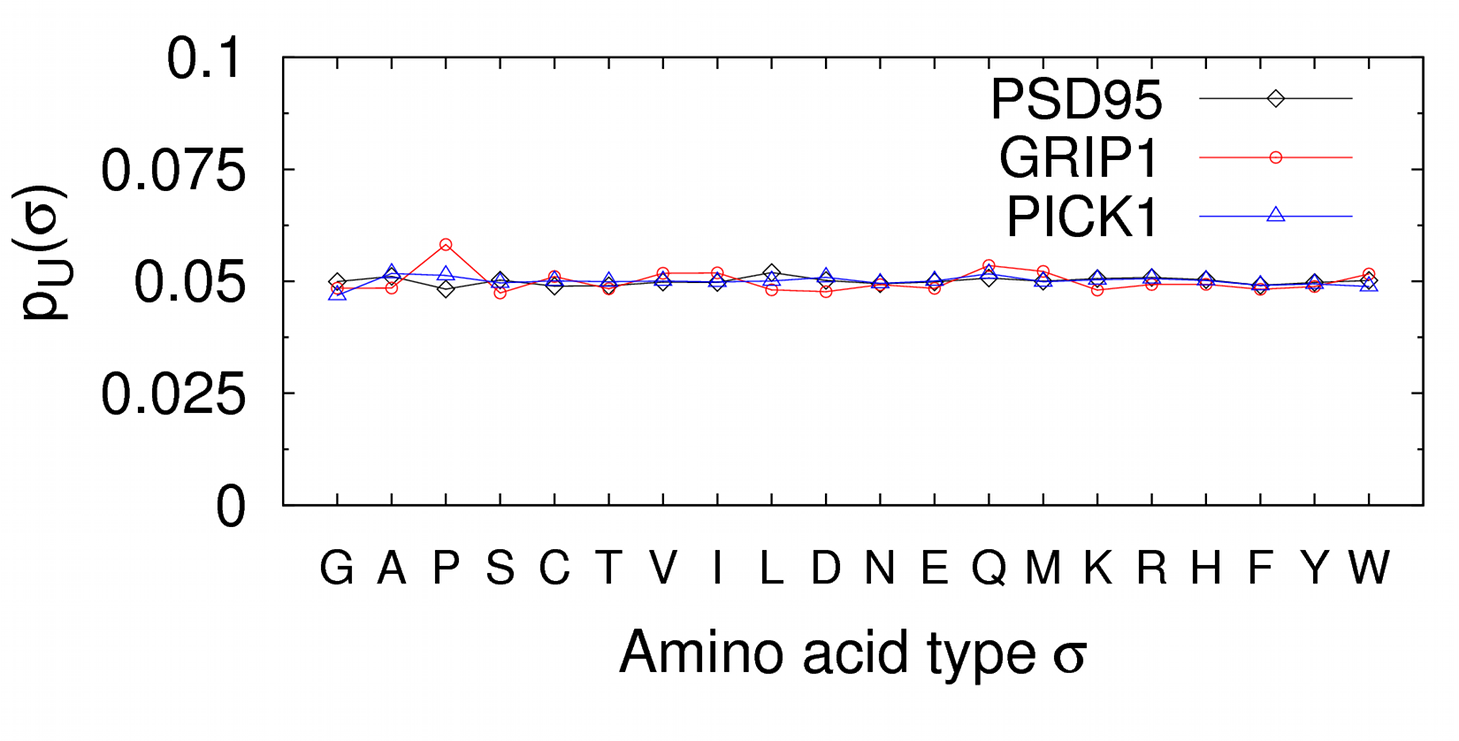

Supplement: Figure S2 — Multisequence Monte Carlo simulations of free peptide chains. The first step of the peptide screening strategy (see Figure 1A) requires obtaining a uniform distribution of sequences, i.e., . Multisequence simulations were performed of the isolated peptides KKE- (PSD95), ATVRT- (GRIP1), and ES- (PICK1), where represents a variable amino acid position, at , , and , respectively. The figure shows probability distributions in amino acid type taken over all 3 variable positions. To achieve roughly flat distributions, i.e., , sets of 20 parameters were determined separately for each peptide by an iterative procedure, as explained in the text. (TIFF) [file pcbi.1003277.s002.tiff]

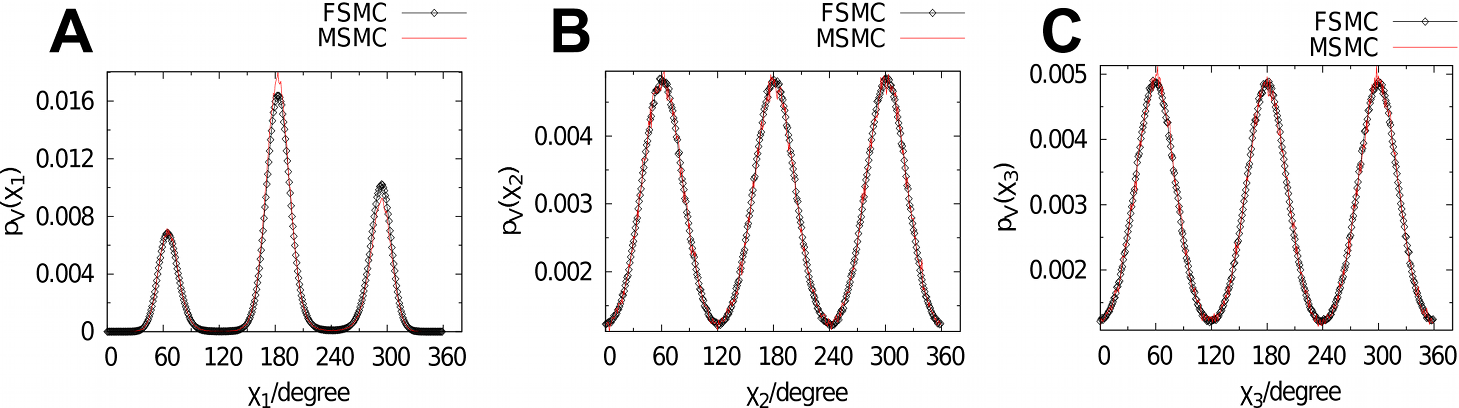

Supplement: Figure S3 — Detailed balance in all-atom multisequence Monte Carlo simulations. To test the soundness of the proposed method, we performed multisequence simulations of the peptide A--A, where is a variable amino acid position, at . These simulations amounts to calculating the thermodynamic behavior for the 20 different variants in a single run. For comparison, therefore, we perform ordinary MC simulations separately for each of the 20 tripeptides AGA, AAA, AVA, etc., at the same temperature. We test the consistency of the two set of results by comparing the probability distributions for different sidechain rotamer angles, . The figure shows for (A) , (B) , and (C) for valine, obtained from the “fixed-sequence” simulation (FSMC) of the tripeptide AVA and the multisequence MC simulation (MSMC) of A--A, with . The consistency of the results confirms that the multisequence simulation samples the correct thermodynamic distribution. (TIFF) [file pcbi.1003277.s003.tiff]

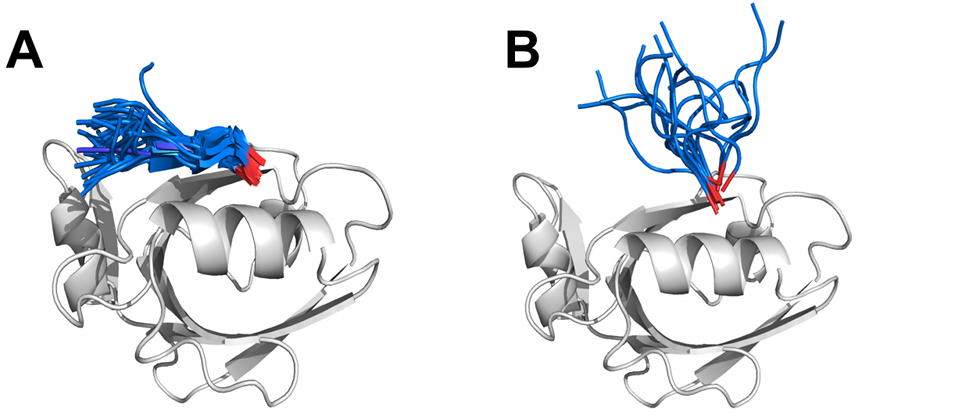

Supplement: Figure S4 — Conformational diversity in the PSD95 peptide-bound state. Superposition of bound conformations from peptide screening simulations of PSD95, sub-grouped into peptides conformations with (A) and (B) 3 Å<RMSD<6 Å, respectively. (TIFF) [file pcbi.1003277.s004.tiff]
